# Supplementary material for: Effect of Oral Health Education Using a Mobile App (OHEMA) on the Oral Health and Swallowing-Related Quality of Life in Community-Based Integrated Care of the Elderly: A Randomized Clinical Trial
Source: Int J Environ Res Public Health. 2021 Nov 7;18(21):11679. doi: 10.3390/ijerph182111679 (PMC8582748; doi:10.3390/ijerph182111679)
Supplement: Supplementary file 1 [file ijerph-18-11679-s001.zip › ijerph-1433009-supplementary.pdf]

International Journal of
Environmental Research and Public Health

MDPI

Supplementary Materials

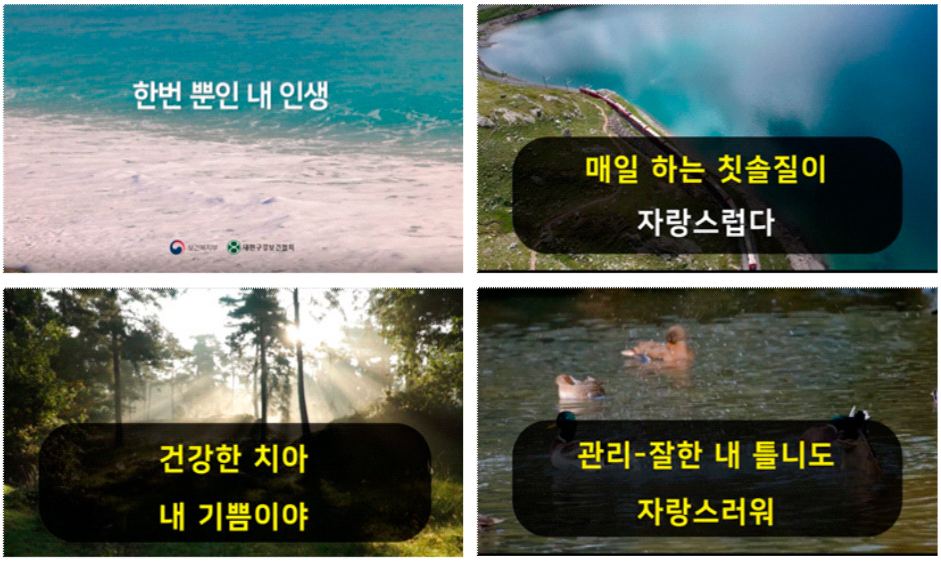

Figure S1. Oral health education trot song. Oral health education trot songs for the elderly were used as an ice breaker before the start of the education program and to form a rapport with the participants. The lyrics of the song were about the oral healthcare method of the elderly, and were intended to motivate the participants. Availavle from: http://www.dental.or.kr/dental_data3.php

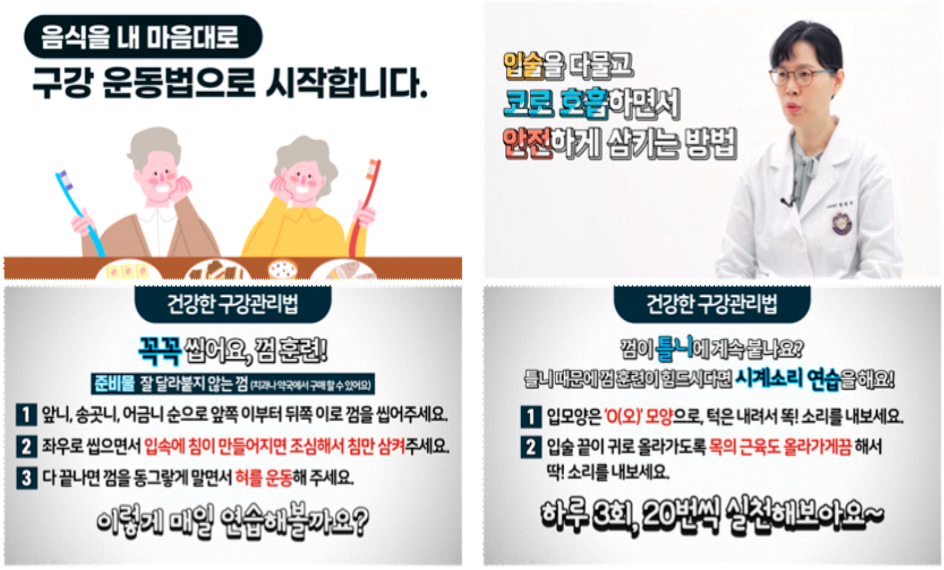

Figure S2. Oral exercise. Oral exercise education consists of gum oral exercise and clock sound exercise. Gum oral exercise is a chewing movement using Xylitol grin gum (Lotte Co. Ltd., Tokyo, Japan). It not only improves the function of teeth, but also induces salivation to relieve dry mouth. The tongue strength and oral muscle function were improved through the clock sound exercise. By sounding the clock while maintaining the correct shape of the mouth, muscle strength around the mouth, including the tongue musculature was improved. Availavle from: http://www.dental.or.kr/dental_data3.php

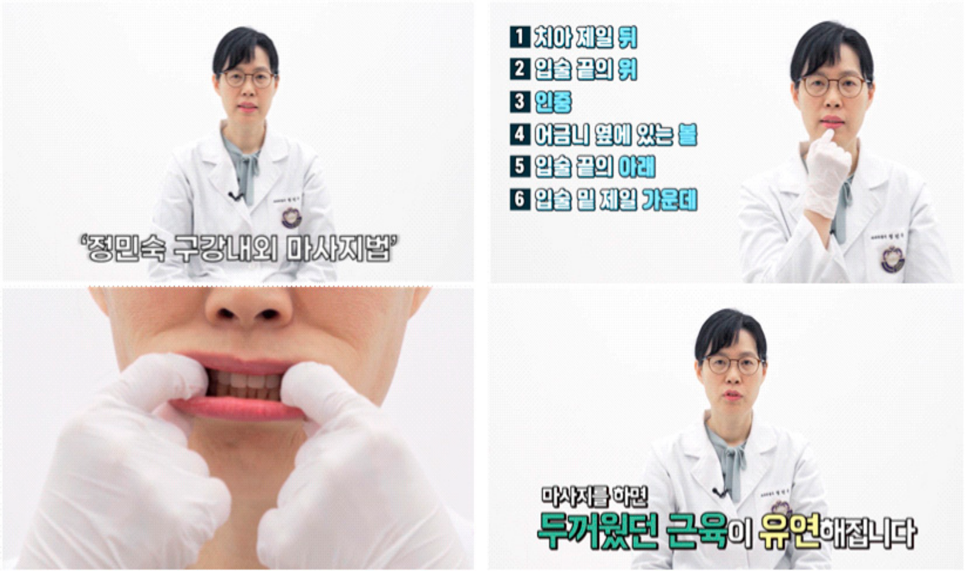

Figure S3. Intraoral and extraoral massage. It stimulates intraoral and extraoral muscles and salivary glands at the same time to increase salivary flow rate and relieve dry mouth. For the maxilla, the thumb is placed intraorally and the index finger extraorally. Then, the thumb and index finger are held together, and light massage is performed. For the mandible, thumb is placed extraorally and the index finger intraorally and light massage is performed in the same way. Finally, the gums are massaged by sweeping the buccal and lingual surface of the gingiva. Availavle from: http://www.dental.or.kr/dental_data3.php

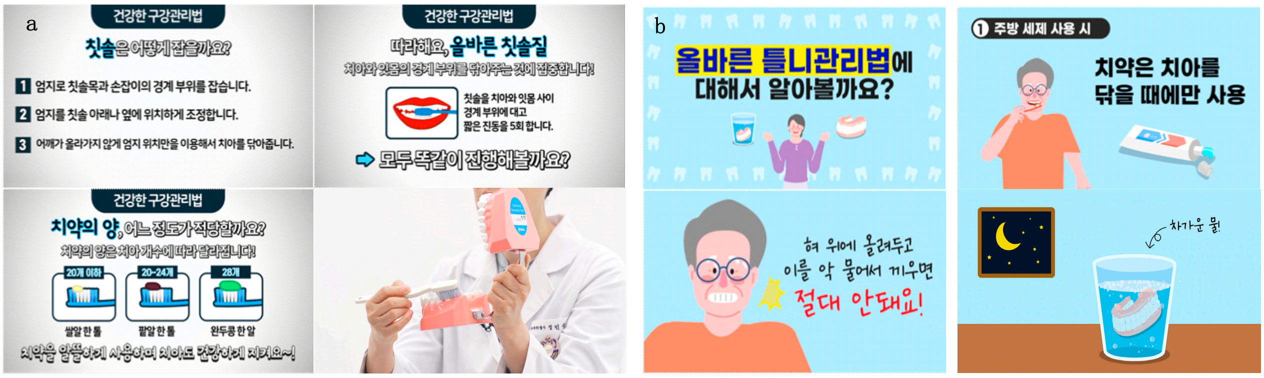

Figure S4. Customized oral hygiene intervention. a) For those who have natural teeth, information is provided on how to select a toothbrush, the appropriate amount of toothpaste, and how to brush their teeth. b) Denture wearers are educated on how to clean and store dentures, and precautions for denture use. Availavle from: http://www.dental.or.kr/dental_data3.php

a

<Self-Oral Healthcare Checklist>

Date : ~

Name : ( )

Date	/	/	/	/	/	/	/
Intraoral & Extraoral massage							
Oral exercise (gum)							
Oral exercise (Clock)							

b

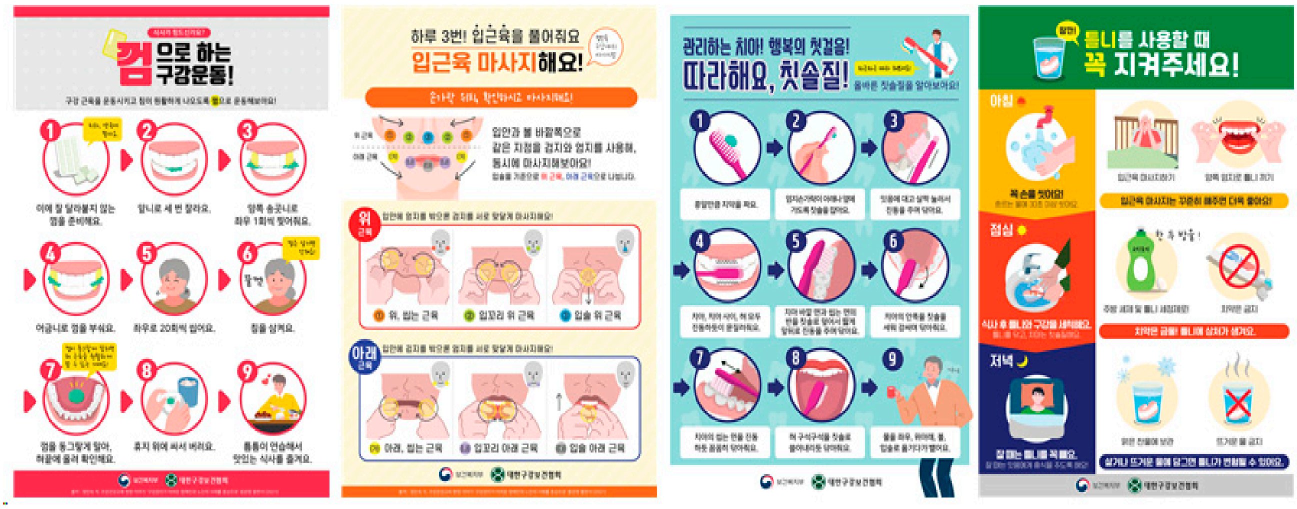

C

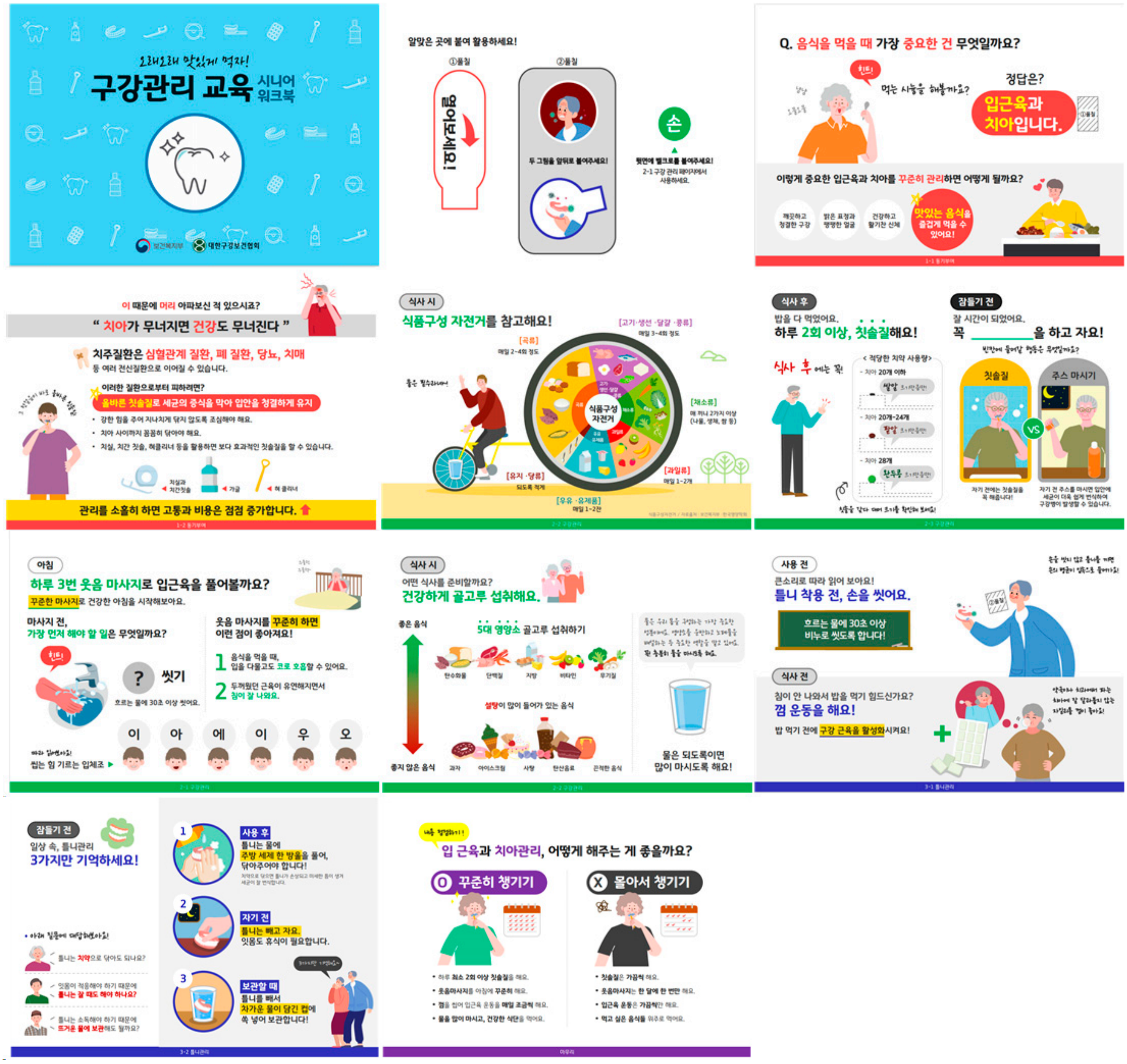

Figure S5. Self oral health care. After each training was completed, a self-checklist was distributed to the participants so that they could put into practice the contents of the training. In addition, the understanding of education was checked through posters and workbooks that included all the educational contents. **a)** Self-checklist, **b)** Poster, **c)** Workbook. Availavle from: http://www.dental.or.kr/dental_data3.php
